# Lymphangioma Circumscriptum (Microcystic Lymphatic Malformation): Palliative Coagulation Using Radiofrequency Current

**DOI:** 10.4103/0974-2077.44165

**Published:** 2008

**Authors:** Omprakash HM, Rajendran SC

**Affiliations:** *Consultant Dermatologist, National Skin and Hair Care Clinic, Bangalore, Karnataka, India*

**Keywords:** Lymphangioma circumscriptum, microcystic lymphatic malformation, radiofrequency current

## Abstract

Lymphangioma circumscriptum (LC, microcystic lymphatic malformation), a hamartomatous lymphatic malformation, is a difficult condition to treat. Different treatments such as surgical excision, lasers, sclerotherapy etc have all been tried with varying success. We report here the efficacy of a radiofrequency current in two patients with lymphangioma circumscriptum. The radiofrequency technique is a safe, economical, and commonly available technique for the treatment of LC; the surgical safety and outcome were satisfactory in our patients.

## INTRODUCTION

Lymphangioma circumscriptum (LC) or microcystic lymphatic malformation is a hamartomatous malformation[1] of the lymphatic channels of the skin. This benign ectasia has two components: (a) the clinically obvious, dermal vesicular component, and (b) the not-so-obvious deeper subcutaneous cisternal element. According to Whimster,[2] the difficulty in treating LC arises from the fact that the subcutaneous muscle-coated lymphatic cisterns receive lymphatic flow from the surrounding tissue, but this is not drained to the normal lymphatic system. These dilated cisterns are pulsating and conduct the lymph through communicating channels into the dermal thin lymphatics, which balloon out into the epidermis.

The definite treatment remains, to this day, radical surgery.[3] However, this may not be practical in many patients because of the risks of anaesthesia, hospitalisation, scarring, and expense. Until a safer nonmutilating, definite therapeutic approach is found, palliative interventions seems to be the only option for many patients. These include sclerotherapy using hypertonic saline,[4] electrocautery, electrofulguration, laser- and light-based device ablation using CO_2_ in continuous or pulsed modes,[5] diode laser with radiofrequency current,[6] pulsed dye laser, and intense pulsed light.[7]

Radiofrequency current devices have replaced electrocautery devices as they are more versatile due to their various therapeutic waveforms: cutting, cutting and coagulating, purely coagulating, and fulguration. We describe here two cases which were followed up for six months after palliative ablation of LC using the coagulation waveform of radiofrequency current.

## CASE REPORTS

Method: A radiofrequency generator – Elmann Surgitron™, model-FFDF.EMC, New York, USA was used for the case series. It generates a 3.8 MHz frequency with a peak power output of 140 Watt ± 20% in a continuous mode. A unipolar copper ball electrode measuring 2 mm was used during surgery. Power of 50 Watts was delivered for 2–3 seconds intermittently by pressing the foot switch, in the coagulation mode of the waveform.

### Case 1

**Figure 1:**
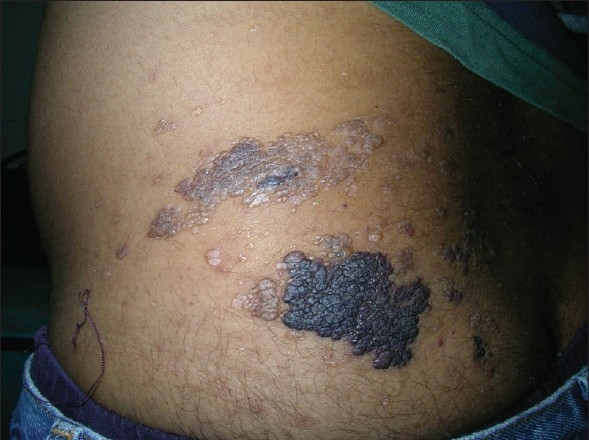
Vesicles containing clear to blood-tinged fluid in left flank (case 1)

A 29 year-old male with Fitzpatrick skin type V presented to our clinic with two fluid-filled lesions in groups on the left flank, the lesions had been prevalent since his childhood. Apart from the cosmetic defect, the main concern of the patient was the watery discharge and intermittent fever. Examination revealed vesicles containing clear to blood-tinged fluid [Figure 1]; the diagnosis was LC. As the patient declined radical surgery, radiofrequency current coagulation was offered as the outpatient surgical option and the need for repeat ablation was explained in case of recurrence.

The lesion was occluded with EMLA cream for one hour prior to surgery. During coagulation, the active electrode ruptured the vesicle when it came in contact with it, producing a popping sound. The electrode was activated for 2–3 seconds until a grayish discoloration of the lesional skin was observed; the perilesional normal skin was also subsequently coagulated.

**Figure 2:**
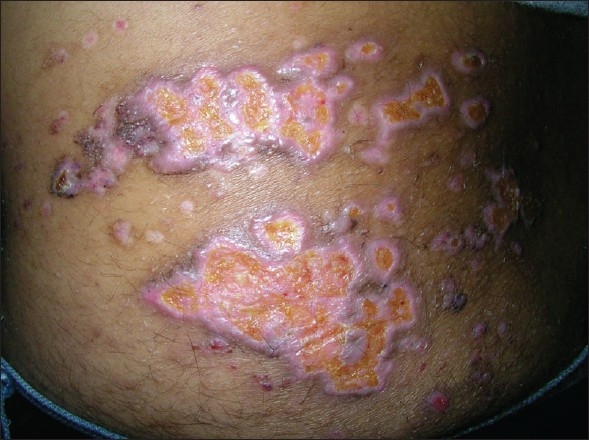
Left flank, three weeks postcoagulation

**Figure 3:**
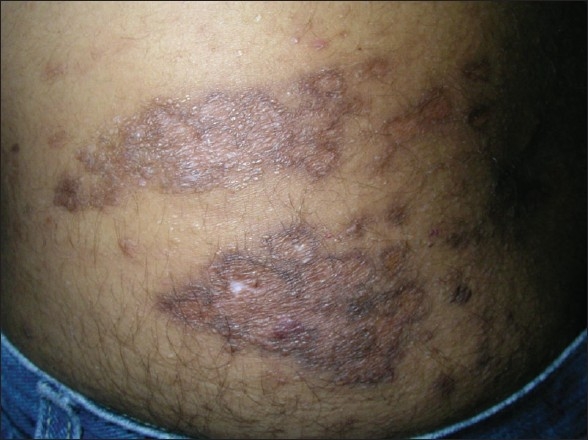
Left flank, six months postcoagulation

The wound was dressed using a sterile paraffin pad but the watery discharge continued for a week. The resulting wound took nearly three weeks to epithelialise [Figure 2]. On follow-up at six months, a hyperpigmented atrophic scar was visible without any discharge or pain [Figure 3] and the patient had not suffered any recurrences during the follow-up period.

### Case 2

**Figure 4:**
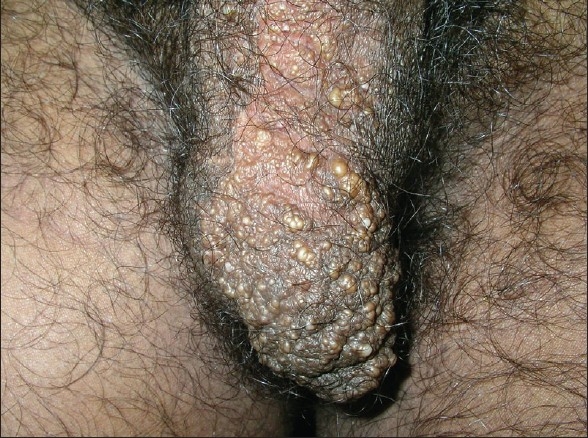
Tiny vesicles on the left side of the scrotum in case 2

A 25 year-old unmarried male with Fitzpatrick skin type V presented with fluid-filled lesions which oozed clear fluid on the left side of the scrotum since the last six months. This fluid soaked his undergarments and affected his quality of life and profession. The lesion was first noticed when he was 15 years old and was surgically excised with primary closure of the wound. He was symptom-free for nearly seven years, only to notice fresh lesions since the last six months months. On examination, multiple, whitish, isolated vesicles were noted on the left side of the scrotum [Figure 4]. The differential diagnoses of lymphangioma circumscriptum, secondary lymphangioma (possibly due to infections such as filariasis or sexually transmitted infections) were considered. History, detailed examination, and serology ruled out filariasis and sexually transmitted disease.

Histopathological exmaination confirmed the diagnosis of LC. Various treatment options were explained to the patient and radiofrequency current coagulation was chosen with his consent.

**Figure 5:**
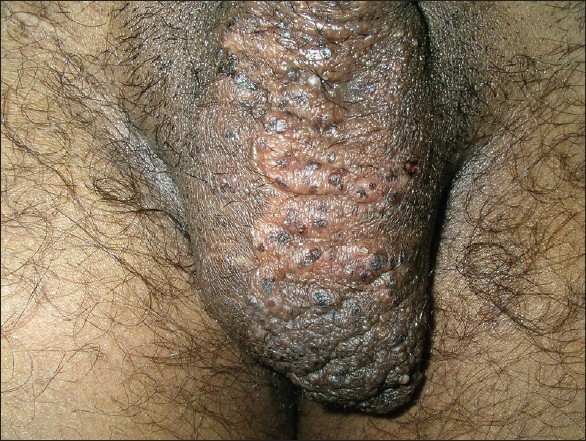
Scrotum, first postoperative day

**Figure 6:**
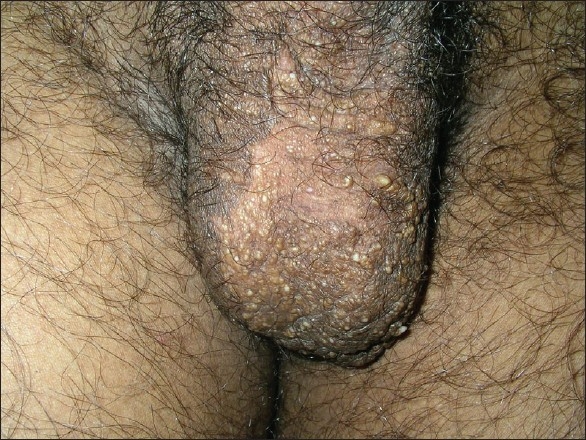
Scrotum, six months postcoagulation

**Figure 7:**
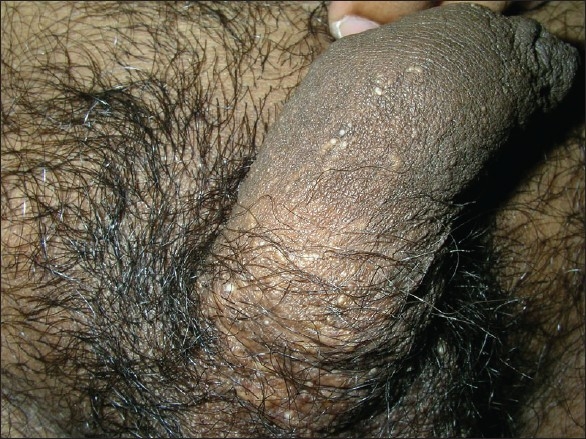
New vesicular eruption on the penis

Under nitrous oxide anaesthesia, the vesicles were touched with the active ball electrode intermittently for 2–3 seconds [Figure 5]. This was done to prevent channeling of the radio waves along the spermatic cord. After the procedure, paraffin dressing was done and systemic antibiotics were started. In comparison to the previous case, the wound epithelialised in a week’s time. After one month, a few vesicles were noted, but the lymphorrhoea was absent. This was possibly because some of the lesions could not be visualised on the operation table with the patient in a supine position as they had emptied in the absence of gravity and hence, coagulation was incomplete. Over the next five months, a few more vesicles were observed with intermittent lymphorrhoea [Figure 6]. During this visit, a few vesicles were noted for the first time on the base of the penis [Figure 7] which prompted a magnetic resonance imaging (MRI) evaluation.

**Figure 8:**
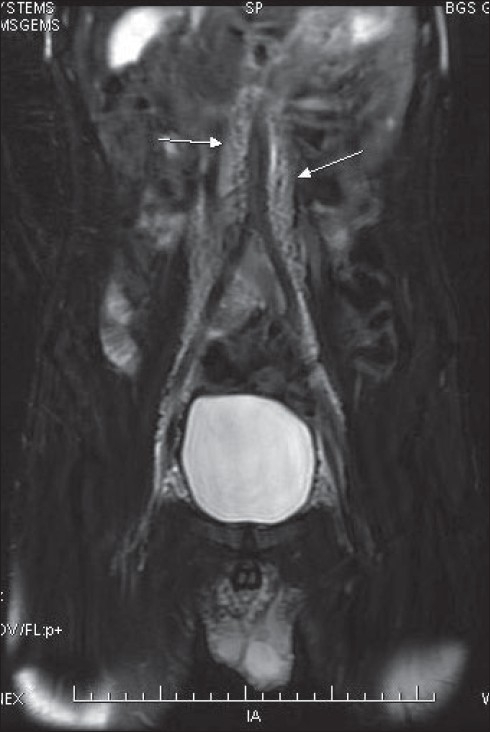
MRI scan of the abdomen and pelvis reveals paraaortic lymphangiectasia (arrow)

**Figure 9:**
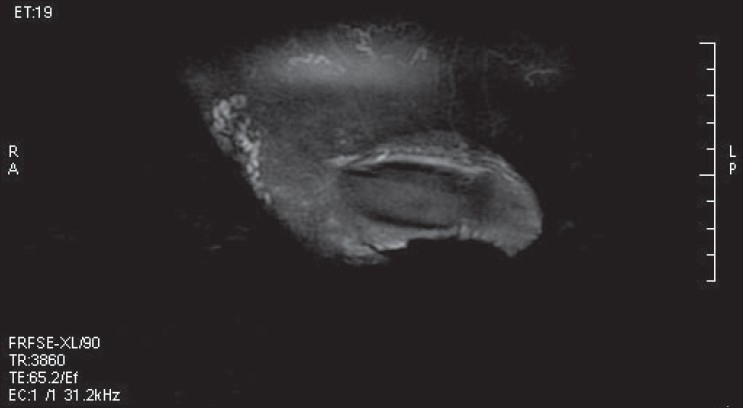
MRI scan of the penis showing lymphangiectasia

MRI of the pelvis and the abdomen revealed multiple, dilated, lymphatic channels in the pelvis and the retroperitoneum extending up to the renal hilum superiorly, with similar smaller channels in the inguinal regions and the glans penis [Figures 8 and 9]. However, there were no mass lesions in the abdomen or the pelvis to account for the lymphectasia. A vascular surgeon’s opinion was sought, but a radical surgical option was ruled out due to the extent of the problem and communication with the normal lymphatics. Further repeat radiofrequency wave coagulation is being contemplated.

## DISCUSSION

Normal skin has no lymphatic channels in the papillary dermis. However, in LC, we find dilated channels in the dermis, which sometimes protrude above the skin surface.[9]

Whereas Case 1 in our series is a typical LC, case 2 is a penoscrotal lymphangioma–an unsual entity of which only around ten cases have been reported to date.[8]

As mentioned earlier, several modalities have been reported for palliative management. A carbon dioxide (CO_2_ ) laser is commonly used in pulsed or continuous mode as it can have an ablative depth of 30 µ and a coagulation depth of 0.6–1.3 mm into the skin,[10] thus, reaching the papillary and partially, the reticular dermal lymphatics required for palliative coagulation. However, a CO_2_ laser may not be available in many centres.

With regards to radiofrequency current, the estimated depth of penetration of radiowaves is equal to half the size of electrode.[11] With our 2 mm ball electrode, the coagulation depth would be roughly 1 mm. Thus, the radiofrequency current used in our patients is almost similar to the CO_2_ laser with regards to tissue interaction and ablative depth.

In our first case, the intervention has produced near-complete clinical ablation with coagulation of lesional and perilesional skin to produce fibrosis of the perivesicular lymphatics. The isolated vesicles have healed without scarring, but the large cluster of vesicles has resolved to leave an atrophic scar. In the second case, however, we have achieved only partial remission with no scarring. The abdominal lymphangiectasia communicating with the scrotal lesion accounts for the relapses and the incomplete remission.

The experience in our patients shows the versatility and effectiveness of radiofrequency machines. They can safely be used in situations where a CO_2_ laser is not available, and they also have the advantage of economy of cost.
